# Molecular characterization of eliminated chromosomes in Hessian fly (*Mayetiola destructor* (Say))

**DOI:** 10.1007/s10577-023-09718-8

**Published:** 2023-01-24

**Authors:** Yan M. Crane, Charles F. Crane, Sue E. Cambron, Lucy J. Springmeyer, Brandon J. Schemerhorn

**Affiliations:** 1 USDA Crop Production and Pest Control Research Unit, West Lafayette, IN 47907 USA; 2 Department of Entomology, Purdue University, West Lafayette, IN 47907 USA; 3 Department of Botany and Plant Pathology, Purdue University, West Lafayette, IN 47907 USA

**Keywords:** Hessian fly, chromosome elimination, supernumerary chromosome, germline, DNA sequencing

## Abstract

Like other cecidomyiid Diptera, Hessian fly has stable S chromosomes and dispensable E chromosomes that are retained only in the germ line. Amplified fragment length polymorphisms (AFLP), suppressive subtractive hybridization (SSH), fluorescent in-situ hybridization (FISH), and sequencing were used to investigate similarities and differences between S and E chromosomes. More than 99.9% of AFLP bands were identical between separated ovary and somatic tissue, but one band was unique to ovary and resembled *Worf*, a non-LTR retrotransposon. Arrayed clones, derived by SSH of somatic from ovarian DNA, showed no clones that were unique to ovary. FISH with BAC clones revealed a diagnostic banding pattern of BAC positions on both autosomes and both sex chromosomes, and each E chromosome shared a pattern with one of the S chromosomes. Sequencing analysis showed that E chromosomes are nearly identical to S chromosomes, since no sequence could be confirmed to belong only to E chromosomes. There were a few questionably E-specific sequences that are candidates for further investigation. Thus, the E chromosomes appear to be derived from S chromosomes by the acquisition or conversion of sequences that produce the negatively heteropycnotic region around the centromere.

**Supplementary Information:**

The online version contains supplementary material available at 10.1007/s10577-023-09718-8.

## Introduction

Species in three dipteran families in the suborder Nematocera possess chromosomes that are maintained in the germ line and eliminated from the soma (Bauer and Beerman 1952; Goday and Esteban 2001; Staiber 2006; White 1973). These chromosomes are referred to as “K” (Keimbahn) chromosomes in the Chironomidae, “L” (limited) chromosomes in the Sciaridae, and “E” (eliminated) chromosomes in the Cecidomyiidae. The chromosomes present in both the germ line and the soma of all three families are referred to as “S” (somatic) chromosomes. Among the germ line-limited chromosomes in these families, the nine K chromosomes of the chironomid *Acricotopus lucidus* have been the most thoroughly characterized (Staiber 2004, 2006; Staiber and Schiffkowski 2000). Each K is composed of large S-homologous sections and K-specific centromeric heterochromatin (Staiber 2002; Staiber 2004; Staiber and Schiffkowski 2000; Staiber and Wahl 2002). Thus, it appears that endopolyploidization, rearrangement, and the accumulation of germ line-specific repetitive sequence account for the evolution of the K chromosomes (Staiber and Schiffkowski 2000). However, it is still unknown whether the L chromosomes of sciarids or the E chromosomes (Es) of cecidomyiids evolved in the same way.

Hessian fly (*Mayetiola destructor*) belongs to the family Cecidomyiidae in the insect order Diptera (flies). It is an important pest of cereal grains in the grass tribe Triticeae, especially bread wheat (*Triticum aestivum* L.). Like other cecidomyiids, Hessian fly has stable S chromosomes and dispensable E chromosomes that are retained only in the germ line. Characterizing Hessian fly E chromosomes might help to elucidate the properties and origin of these dispensable chromosomes in all cecidomyiids. Hessian fly genomic organization resembles that in the majority of non-pedogenetic cecidomyiids (Stuart and Hatchett 1988). Its S genome consists of four chromosomes, including two autosomes (A1 and A2) and two X chromosomes (X1 and X2). The female somatic karyotype is diploid for all four S chromosomes (A1A2X1X2/A1A2X1X2). The male soma is diploid for the autosomes but contains only the maternally derived X chromosomes (A1A2X1X2/A1A2OO). The germ line of both sexes is diploid for the S chromosomes and contains a variable number (~32) of E chromosomes. At or before the fifth cleavage division of embryogenesis, all the E chromosomes are eliminated from the presumptive somatic nuclei. The paternally derived X chromosomes are also eliminated if the embryo is to develop as a male (Shukle and Stuart 1993). The Hessian fly E chromosomes, like those of the cecidomyiid *Wachtliella persicariae*, are essential to gonad development and fertility (Bantock 1970; Berger et al., 1966).

Because of its economic importance and its genetic tractability, genomic tools have been developed to investigate Hessian fly biology (Behura et al. 2004; Lobo et al. 2006). These include Hessian fly genomic bacterial artificial chromosomes (BACs), which can be used in fluorescence in situ hybridization (FISH) experiments to locate specific segments of genomic DNA on Hessian fly chromosomes. In the present investigation, Hessian fly BACs were used in FISH experiments to determine whether Hessian fly E chromosomes and S chromosomes share specific segments of DNA. Previous work (Schemerhorn et al. 2009) had identified microsatellite markers that reside on 170 Hessian fly BACs and mapped 94 of them to polytene S chromosomes. Also, amplified fragment length polymorphisms (AFLPs) were used to search for sequence differences between tissues that did or did not possess E chromosomes. In addition, a genomic library was prepared after subtraction of sequences common to all tissues, including S chromosomes and any sequences that are the same in S and related E chromosomes. Arrayed clones from this library were hybridized with probe DNA from ovary or from the soma, to search for clones specific to the germline. Finally, DNA was extracted separately from heads and ovaries, Illumina sequenced, and searched for sequences specific to ovaries. None of the preceding methods identified sequences that unequivocally belong to E chromosomes, although several candidates were identified that might reside only on E chromosomes. Therefore, the E chromosomes of the Hessian fly, like the K chromosomes of *A. lucidus*, apparently have evolved by germ-line-specific duplication or nondisjunction of the S chromosomes of Hessian fly or a related species.

## Materials and methods

### Sources of Hessian flies and genomic DNA

Hessian fly strain (biotype) L was maintained in the USDA-ARS greenhouse facility at Purdue University, West Lafayette, Indiana, USA, according to the methods of Gallun (1961). The sampled flies were progeny of a single mated female on a single, isolated wheat plant. Ovaries (Suppl. Fig. 1) were the source of E chromosome DNA, and testes were used for fluorescent in situ hybridization (FISH). Gonads were dissected from 19 to 24-day-old third instar larvae in 50% Ringer’s solution under an Olympus SZX16 dissecting microscope, or alternatively the potted host plant was stored at 4 °C up to 3 weeks to stop development. After dissection, the ovaries were collected in bulk (Suppl. Fig. 2) on dry ice and stored at −80 °C. The heads of male biotype L were collected and pooled as the somatic tissue, known to be free of E chromosomes. Heads were collected separately from the ovaries, frozen on dry ice, and stored at −80 °C. DNA was isolated from ovaries and heads separately using the DNeasy Tissue Kit (catalog number 69506, Qiagen Inc., Valencia, CA), as described by Schemerhorn et al. (2008).

### Slide preparation for fluorescent in situ DNA hybridization (FISH)

Testes were collected from 19 to 21-day-old male 3rd instar larvae (whose head has turned) in half-strength Ringer’s solution (250 μl) in microcentrifuge tubes. The testes were briefly spun down at low speed in a microcentrifuge to collect testes at the bottom. The supernatant was removed and replaced with 250 μl of 3 ethanol: 1 acetic acid, allowed to stand 10 min, and then spun at >500 g for 1 min. The solution was removed and allowed to air dry enough to remove the ethanol, but not dry the testes. Three drops (ca. 150 μl) of 50% acetic acid were added to the tube for every 5 testes there. The cell mixture was dropped onto clean slides and dried overnight.

### DNA probes and FISH

The DNA probes used in this study are shown in Supplemental Table 1 (Schemerhorn et al. 2008). The FISH protocol was modified from Schemerhorn et al. (2009) for simultaneous hybridization with four differently labeled probes. DNA probes were prepared from amplification product of microsatellite primers using BAC DNA as the template. Probes were labeled by nick-translation (kit from Roche Applied Science, Penzberg, Germany) with dUTP conjugated with biotin, digoxigenin, or fluorescein (all from Invitrogen, Waltham, MA). The hybridization mix and conditions exactly followed Schemerhorn et al. (2009). Biotinylated probe sites were detected with streptavidin, amplified with biotinylated anti-streptavidin, and coated with a second layer of streptavidin that is conjugated with Alexa Fluor 647 (Invitrogen), which fluoresces blue. Simultaneously, sites of digoxigenin-labeled probe hybridization were detected with mouse anti-digoxigenin and amplified with anti-mouse antibody that is conjugated with Alexa Fluor 568 (Invitrogen), which fluoresces red. Fluorescein was detected with anti-fluorescein conjugated with Oregon Green (Invitrogen), and amplified with goat IgG fraction conjugated with Alexa Fluor 488 (Invitrogen), which fluoresces green. The single probe that had been labeled with both digoxigenin and fluorescein fluoresced red and green and thus appeared yellow. After washes to remove unbound probe, the slides were mounted with Vectashield antifade (Vector Labs, Newark, CA). Chromosomes were photographed with a Nikon Eclipse E400 microscope equipped for epifluorescence with appropriate bandpass filters and an Olympus digital microscope camera. Digital images were acquired and contrast-enhanced using Adobe Photoshop CS5.

### Amplified fragment length polymorphisms (AFLP)-PCR

Amplified fragment length polymorphisms were identified using the AFLP® Analysis System II and AFLP® Small Genome Primer Kit (Invitrogen, Carlsbad, CA) according to the manufacturer’s instructions with the following modifications. The selective amplifications were performed using the following restriction enzymes and selective nucleotides: EcoRI-AA/MseI-CAC; EcoRI-AA/MseI-CTA, EcoRI-AA/MseI-CAG; EcoRI-AA/MseI-CAT; EcoRI-TG/MseI-CAC; EcoRI-TG/MseI-CTA; EcoRI-TG/MseI-CAG; EcoRI-TG/MseI-CAT; EcoRI-AG/MseI-CAC; EcoRI-AG/MseI-CTA; EcoRI-AG/MseI-CAG; EcoRI-AG/MseI-CAT; EcoRI-AC/MseI-CAC; EcoRI-AC/MseI-CTA; EcoRI-AC/MseI-CAG; EcoRI-AC/MseI-CAT. The PCR program was one cycle at 94 °C for 30 s; 65 °C for 30 s; and 72 ° for 60 s; followed by a touch down phase of 12 cycles of 0.7 °C lower annealing temperature each cycle. Afterwards, 22 cycles were performed at 94 °C for 30 s; 56 °C for 30 s; and 72 °C for 60 s. The amplifications were carried out in a PTC220 programmable Thermal Controller (Bio-Rad, Hercules, CA). An equal volume of formamide buffer (98% formamide, 10 mM EDTA, pH 8.0) was added to each PCR reaction (20 μl). The samples were denatured by heating at 95 °C for 5 min and then immediately placed on ice. Eighteen μl of each sample were loaded on a 7% polyacrylamide gel [acrylamide:bisacrylamide (20:1); 7.5 M urea; 1X TBE buffer]. Polyacrylamide gel electrophoresis was performed until the xylene cyanol dye band was about 2–3 cm from the bottom of the gel. The gel was treated with 1% nitric acid for 10 min and silver-stained with the protocol of Creste et al. (2001), to detect the AFLP fingerprints.

### Suppressive subtractive hybridization (SSH) DNA libraries

Suppressive subtractive hybridization (SSH) libraries were generated and screened using the PCR-Select cDNA subtraction kit and PCR-Select differential screening kit (Catalog #63791 and #637403, Clontech, Palo Alto, CA), modified upon the advice of the Clontech support staff by using genomic DNA as the starting material. In the SSH library, the DNA from ovaries of female larvae was used as the “tester,” and the DNA from the remaining body parts was used as the “driver” (Diatchenko et al. 1996). After completion of SSH (subtracting “driver” from “tester”), unhybridized DNA was PCR amplified by 25 cycles, instead of the 15 cycles in the protocol, to produce sufficient DNA for cloning. Invitrogen’s TOPO TA cloning kit was used to construct a library from the subtracted-and-amplified DNA. Eight distinct 384-spot DNA macroarrays were made by spotting clones from this library onto nylon filters at the Purdue Genomics Center. Duplicate macroarrays were hybridized with ^32^P labeled genomic DNA extracted from Hessian flies whose ovaries had been removed, or ^32^P labeled genomic DNA extracted from Hessian fly ovaries.

### Sequencing and analysis

In an attempt to identify distinctive sequences that are limited to E chromosomes, paired-end 150-base reads were produced by the Purdue Genomics Core on an Illumina NovaSeq sequencer. In total, 0.62 μg of DNA was sequenced from ovary-containing tissue, and 2.25 μg of DNA was sequenced from heads. Adapter and low-quality ends were trimmed off with Trimmomatic (Bolger et al. 2014). Initial bioinformatic processing was performed by RAPiD Genomics, Inc., Gainesville, FL. Reads were aligned to GenBank accession GCA_000149185 (the “Kansas Great Plain” Hessian fly genome) using bwa mem with k set to 19. Unaligned reads were collected separately for heads and ovaries. The unaligned reads from heads were assembled with SPAdes (Bankevich et al. 2012; Nurk et al. 2013) using 32 cores and 475 Gb of memory, setting the value of k automatically based on read length. Unaligned ovarian reads were aligned to the SPAdes assembly from heads. Ovarian reads that remained unaligned to heads were then assembled with SPAdes as above, resulting in 156,569 contigs that were further analyzed in house.

Contigs were selected for length over 100 bases. These contigs were filtered by blast (Altschul et al. 1997) alignment at 1e-10 to a custom database consisting of the wheat and human genomes plus the whole genomes of bacterial, archaeal, fungal, viral, protozoan, and invertebrate taxa, which were the representative or latest available genomes for these taxa in NCBI genomes (https://ftp.ncbi.nlm.nih.gov/refseq/release/) as of 20 May 2022 (Suppl. Table 2). Contigs that did not align to the custom database were then blastn-aligned at 1e-10 to the Berkeley Hessian fly genome (NCBI accession GenBank GCA_001014435.1), noting the count of sites where each contig hit in the genome. Contigs that hit GCA_001014435.1 exactly once or twice were then blastx-aligned to the NCBI nr protein database. Such contigs that aligned to nr were then checked for taxonomic annotation. Those that aligned to insect taxa were used to design PCR primers with primer3 (Untergasser et al. 2012; Koressaar and Remm 2007) that would amplify subsequences at least 250 bases long from the Berkeley genome. The resulting primers were blastn-aligned to the Berkeley and Kansas Great Plain assemblies at an e-value of 0.1, and the hits were counted as exact or inexact occurrences separately in each genome, depending on the absence or presence of any mismatched nucleotide relative to the primer sequence. Instances were identified where each primer hit only the Berkeley assembly exactly; their amplicons were checked for hits and annotation in GenBank nr. A final screen removed amplicons that also hit any one of the other databases listed in Supplemental Table 2. For each annotated contig, a single primer3-generated primer pair was selected with melting temperatures closest to 60.0 °C.

### Contig confirmation by PCR

There were 388 primer pairs from 368 contigs that were tested with seven families, each consisting of progeny of one male and one female inhabiting one wheat plant. DNA was extracted from ovary and head tissue as described above. Amplification used the MyFi™ DNA polymerase protocol from Bioline (Taunton, MA). The PCR cycle was initial denaturation at 95° for 5 min followed by 35 cycles of 95 °C for 30 s, 30 s with the annealing temperature set 5 °C below the melting temperature of the primer pair, and extension at 72 °C for 60 s, and final elongation at 72 °C for 10 min. The product was stored at 4 °C.

## Results

### FISH

**Fig. 1:**
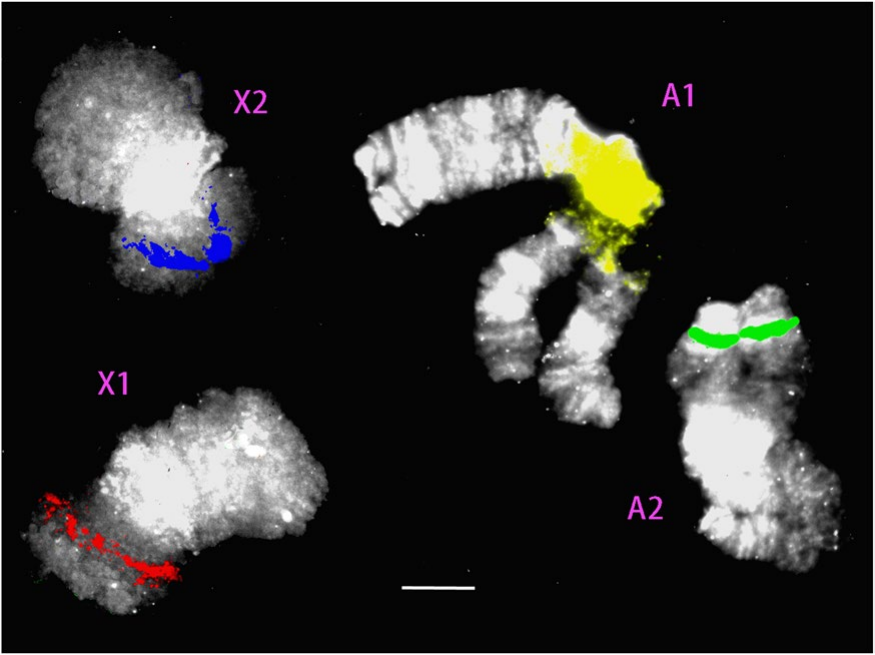
Multiplexed FISH of polytene chromosomes in salivary glands using the chromosome-specific probes listed in Supplemental Table 1. Yellow signal is the nucleolar organizer region (NOR) on chromosome A1. Green signal is on the long arm of chromosome A2. Red signal is on the short arm of chromosome X1. Blue signal is on the short arm of chromosome X2. Bar represents 10 μm

**Fig. 2:**
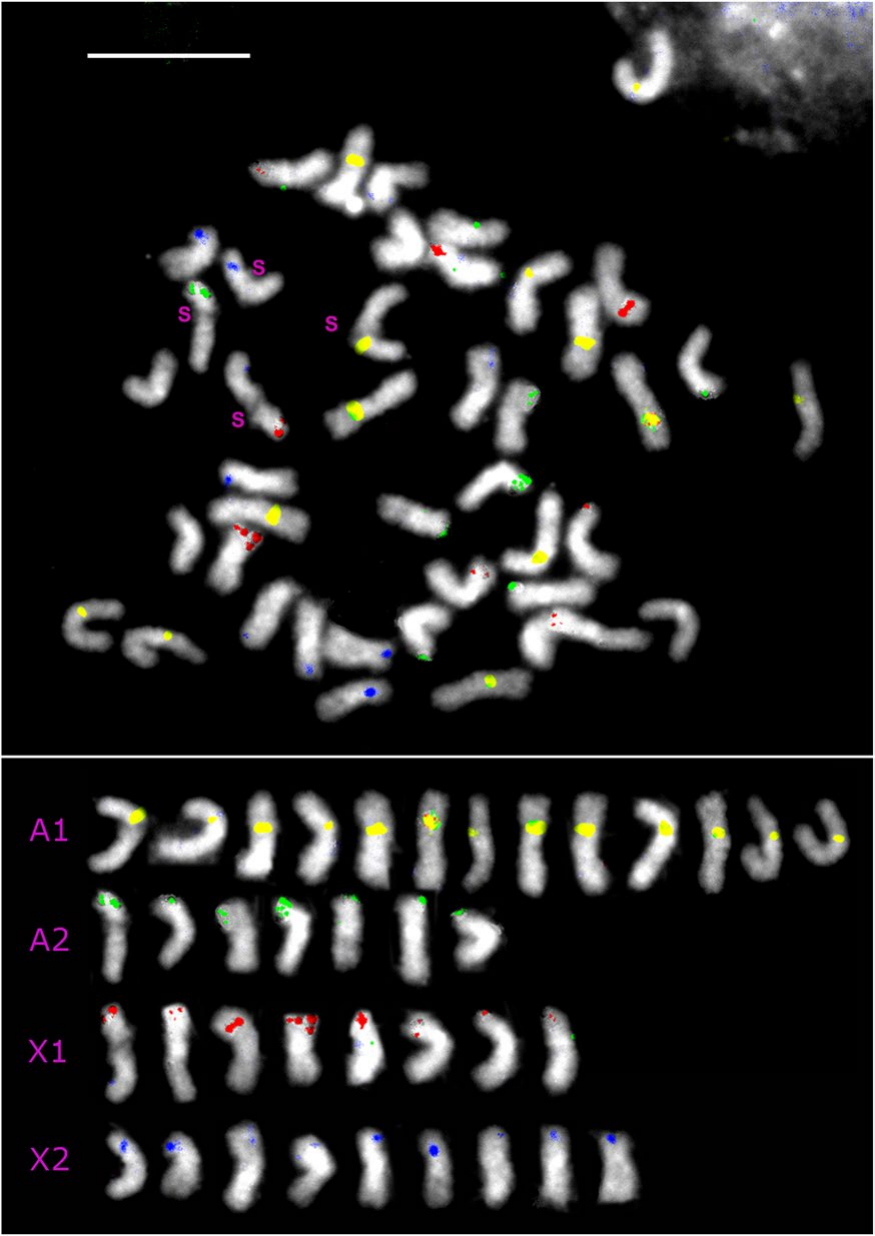
Above, metaphase of testis with 4 maternally derived S chromosomes (labeled s), 33 additional chromosomes with probe signals, and 5 additional chromosomes without signals. The probes and signal colors are the same as in Fig. 1. Below, the 37 chromosomes from above arranged by type of hybridization signal. The 5 chromosomes without signal were not included. Bar represents 10 μm

**Fig. 3:**
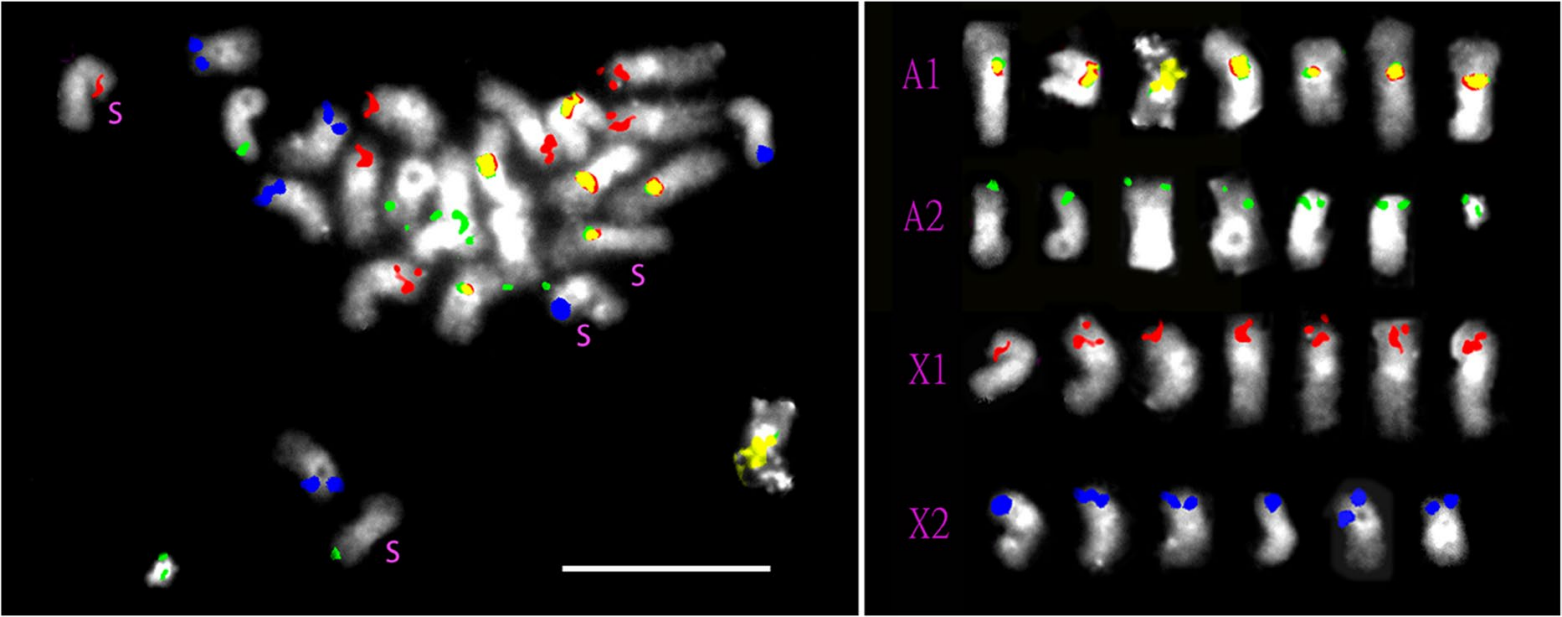
Left, metaphase of testis with 4 maternally derived S chromosomes (labeled s) and 23 additional chromosomes. The probes and signal colors are the same as in Fig. 1. Right, all 27 chromosomes from left sorted by color of hybridization signal. Bar represents 10 μm

Four diagnostic BAC probes (Table 1) were identified that distinguished the polytene A1, A2, X1, and X2 chromosomes in salivary glands when multiplexed for FISH (Fig. 1). The polytene chromosomes could be reliably identified on the basis of total length and arm lengths (Fig. 1), and three of the BACs contained microsatellite markers (Table 1). Metaphase spreads of testis cells contained E chromosomes as well as the S chromosomes, which could be identified because of their distinctive, negatively heteropycnotic centromere in the median position (Stuart and Hatchett 1988). Figures 2 and 3 show two such cells, including the respective chromosomes of these cells sorted by presence of their diagnostic BAC hybridization signals. All the successfully hybridized E chromosomes had a signal, indicating homoeology to the S chromosomes. Furthermore, the number (one) and relative position of probe signals were stable among the chromosomes, indicating that they had not undergone large-scale deletions, duplications, inversions, or translocations. The signals varied in intensity, but it is not known if this variation is reproducible among the E chromosomes, because of plausible experimental variation in probe concentration, detection reagents, exposure and retention of target DNA in the chromosomes, and intensity thresholding in processing the images. Also, the number of E chromosomes varied among cells within and among individual flies. Among the 88 mitotic metaphase cells observed in testes, there were 20 to 46 E chromosomes, in accordance with the upper limit of 45 reported by Rider et al. (2002). The most common E chromosome number was between 24 and 32. The four types of E chromosomes varied independently in number, but the A1 homoeolog (yellow label in Fig. 2) was at least as abundant as any of the three other classes.

**Table 1:** Details of possible E chromosome sequences segregating among families

Locus name	PCR product	Primer	Tm	GenBank accession hits	GenBank annotation
HFE174	330 bp	L: GTGGAAGAACAGTTGCAGCG	60.041	XP_031619250.1	Uncharacterized protein LOC116338260 [*Contarinia nasturtii*]; jerky protein homolog-like [*Epinephelus lanceolatus*]
		R: GCCGGAGATGGTTTGACCTT	60.323	XP_033472895.1	
HFE315	284 bp	L: GGAGGCATTCTCTCCGCAAT	60.179	RYE18558.1	Reverse transcriptase family protein, partial [Sphingobacteriaceae bacterium]; RNA-directed DNA polymerase from mobile element jockey [*Araneus ventricosus*]
		R: TGCGCTTGTTTCCTAGTTCCT	59.928	GBM72862.1

### AFLP

**Fig. 4:**
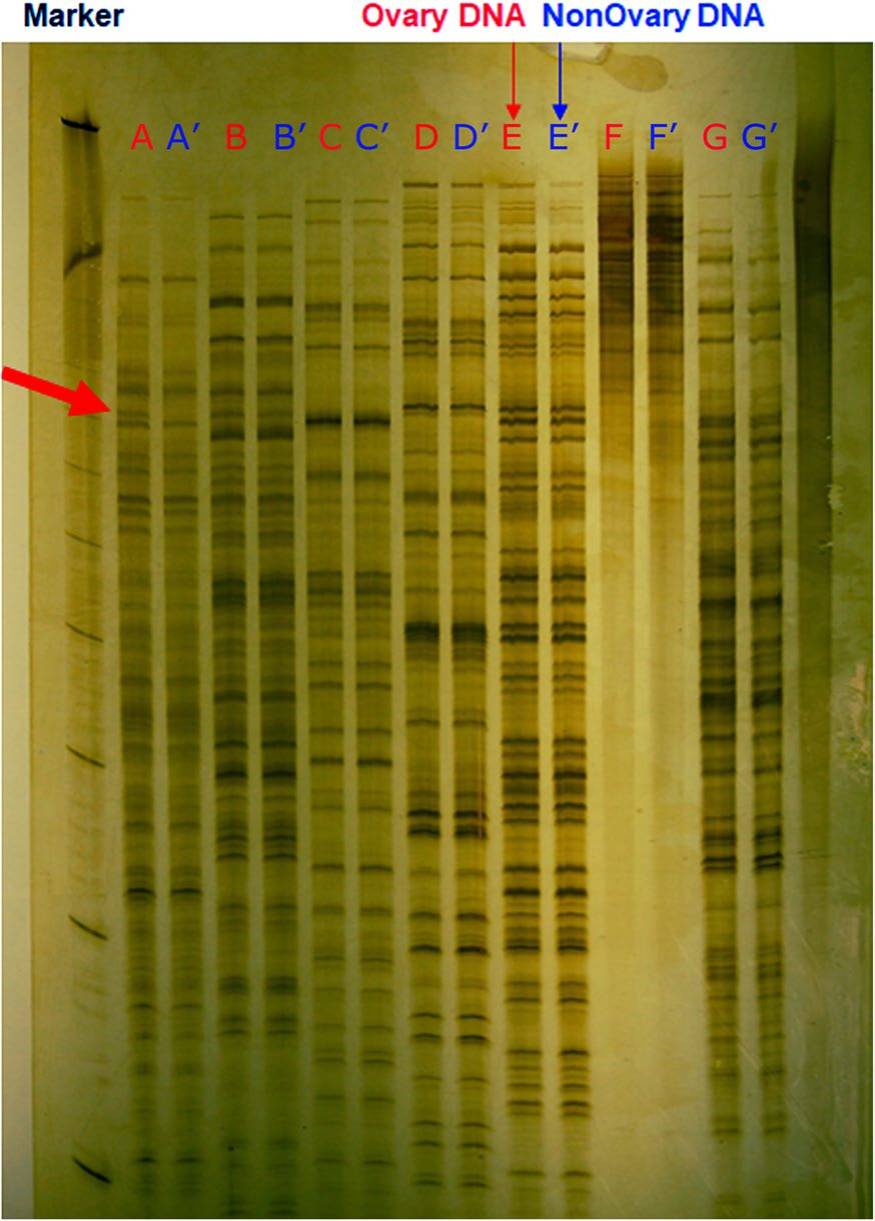
Amplified fragment length polymorphism (AFLP) of ovarian (without superscript) and somatic (with superscript) DNA after digestion with different combinations of restriction endonucleases, E (EcoRI) and M (MseI), and selective bases. Red arrow denotes an extra band present only in ovaries. Leftmost lane is size markers. (A) E-AA/M-CAC, (B) E-TG/M-CAC, (C) E-AG/M-CTA, (D) E-AG/M-CAG (E) E-TG/M-CAG, (F) E-TG/M-CAT, (G) E-AC/M-CTA

A single AFLP band (Fig. 4) was the only size variant noted between ovaries and somatic tissue in more than 1000 AFLP fingerprints that had been generated with 16 primer pairs. Two Sanger sequences, designated E1-1 and E1-2, were obtained from cloning it. Neither E1-1 nor E1-2 (supplemental Table 3) matched the other by blastn at 0.001 e-value. They were aligned with blastn at 0.001 to both *M. destructor* assemblies, versions 12.09 and 24.12 of RepBase (Bao et al. 2015), the subset of 8703 shotgun contigs that matched only the Berkeley genome, and by blastx to the NCBI nr protein database. The alignment to repbase.12.09 indicated that E1-2 is a non-LTR retrotransposon of type Worf (Bachtrog 2003), but the alignment to repbase.24.12 gave no hits for either E1-1 or E1-2. Neither E1-1 nor E1-2 aligned to the subset of 8703 sequences.

Sequence E1-1 hit the Berkeley genome only once, in scaffold JXPD01004305.1, bases 6125 through 6497, at 0.0 e-value and 372/373 identities. Sequence E1-1 hit Kansas Great Plain once, in unplaced scaffold GL503032.1 (151,866–152,238, 0.0, 372/373). Scaffold GL503032.1 (155,982–150,024) mapped to Berkeley scaffold JXPD01023314.1 (2530–7540) at 0.0 e-value and 5180/8185 identities.

Sequence E1-2 hit Berkeley once, in scaffold JXPD01023314.1 (2249–2578, 1e-63, 264/330). Sequence E1-2 hit Kansas Great Plain in three places, one in accession AEGA01024186.1, contig Contig24204 (822–1202, 1e-172, 364/381), another in accession GL502719.1, scaffold Un.19954 (12,541–12,839, 5e-63, 245/301), and finally in accession GL502163.1, scaffold Un.6486 (73,019–73,337, 2e-62, 256/319). GL502719.1 mapped to AEGA01024186.1 at 2e-177 and 364/381 identities.

Sequence E1-1 hit 31 accessions in the nr protein database at 25–30% identity; almost all are nucleic-acid binding proteins from arthropod retrotransposons, and four are annotated as RNA-dependent DNA polymerases. Sequence E1-2 hit 195 accessions in nr at 25–70% identity; most are hypothetical proteins from Diptera, including many cecidomyiid midges, and 16 are annotated as reverse transcriptases, i.e., RNA-dependent DNA polymerases.

Both sequences were also tblastx aligned to the databases in Supplemental Table 2 as well as the SPAdes assembly of shotgun reads. The only hit to any of these databases at *p* < 0.001 was of E1-2 to a locus in *Enterobacter cloacae*, accession NZ_QFLX01000131.1, at 8e-08 with 31% identity and 58% positives.

### SSH

**Fig. 5:**
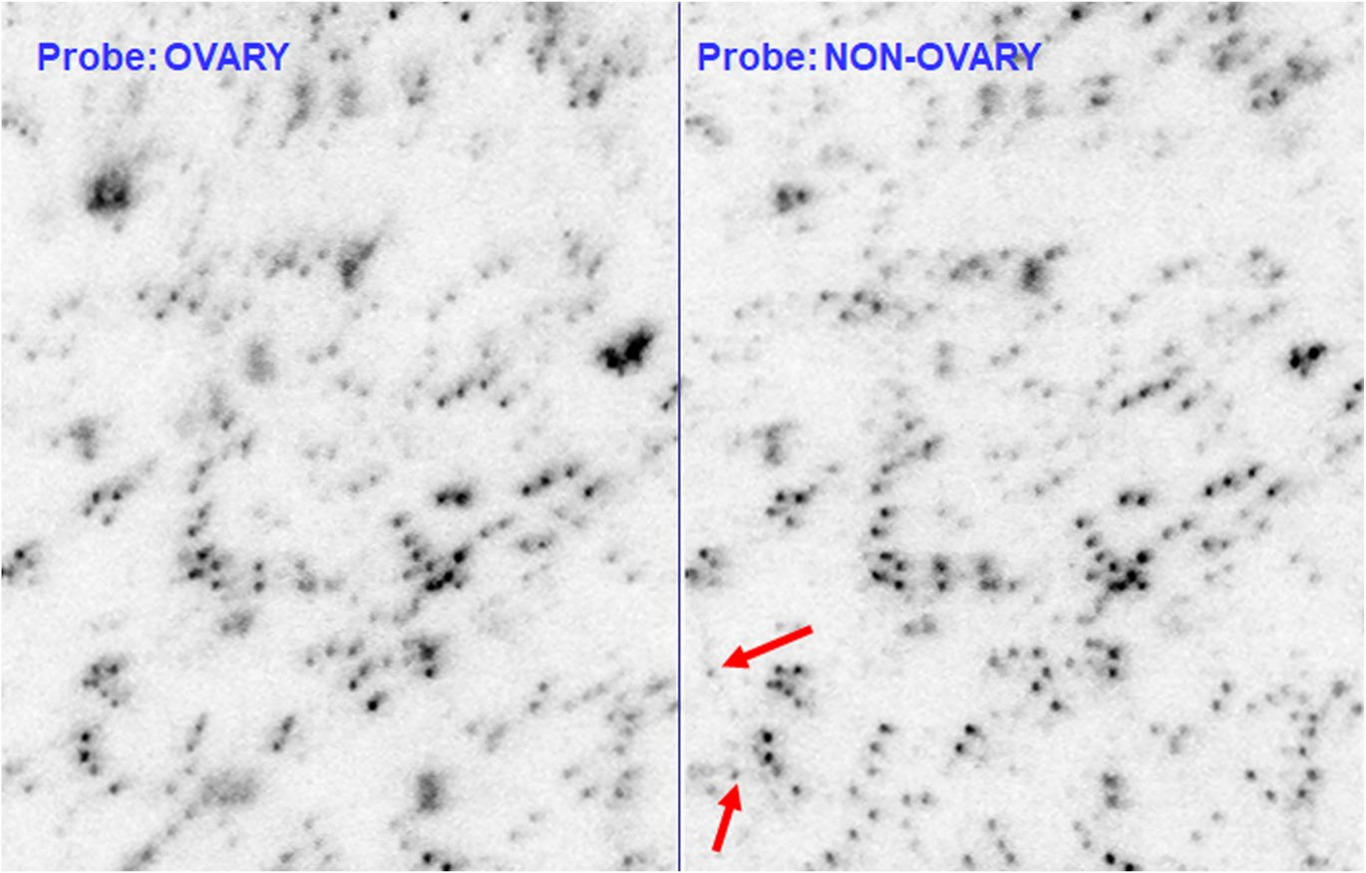
Autoradiograms of duplicate nylon macroarrays hybridized with ^32^P-labeled ovarian DNA (left panel) or somatic DNA (right panel). Red arrows denote extra spots visible with the somatic DNA

About 1/6th of the 3072 arrayed clones appeared on autoradiograms. These clones were present at sufficient copy number in the Hessian fly genome to produce the strongest signals. The hybridized spots were almost identical between the head and ovarian probes (Fig. 5), although there was slight variation in relative intensity among spots. Two extra spots were noted with the somatic (not ovarian) probe (Fig. 5).

### Shotgun sequencing

**Fig. 6:**
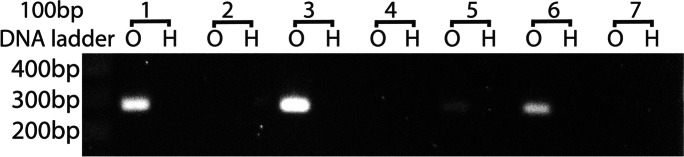
Ethidium-stained agarose gel with amplified product of primer pair HFE315 in ovarian DNA of four of seven families (left lanes of pairs) and not in head DNA (right lanes of pairs)

**Fig. 7:**
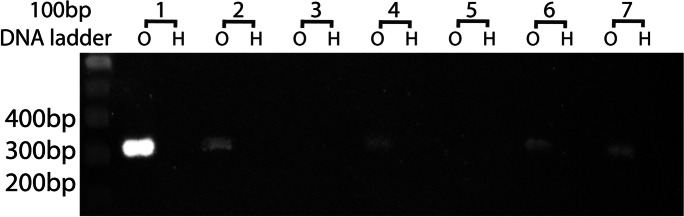
Ethidium-stained agarose gel with amplified product of primer pair HFE174 in ovarian DNA of five of seven families and no lanes of head DNA

Ovaries yielded 2,793,053,864 reads totaling 420,628,274,131 bases, while heads yielded 99,928,596 reads totaling 15,049,348,815 bases. These are nominally 2263.5× and 81.0× coverage of the 185,827,756-base Kansas Great Plain Hessian fly genome, NCBI accession GCA_000149185. The initial SPAdes assembly of ovarian reads consisted of 156,569 contigs, of which 115,712 were at least 100 bases in length. Of these, 46,151 exceeded 300 bases and 14,730 exceeded 1000 bases. There were 2393 contigs that matched the Berkeley genome exactly once for at least 250 bases, and 365 contigs that matched twice for at least 250 bases per hit. Of the former, 193 contigs hit annotated accessions in nr, while of the latter, 27 hit annotation in nr. Primers for these 220 contigs were selected to exist in two sites in the Berkeley genome, and almost all amplified the same product size in both heads and ovaries. Only one pair of primers failed to amplify a product in either heads or ovaries. Two primer pairs (HFE 174 and HFE315) amplified a product in ovaries only, in only four (HFE 315, Fig. 6) or five (HFE 174, Fig. 7) of the seven families. Both HFE174 and HFE315 amplified product in families 1 and 6. HFE174 (contig NODE_15063_length_418_cov_24.005865) hit an uncharacterized protein from the swede midge, *Contarinia nasturtii*, and also was similar to a jerky protein from a fish, *Epinephelus lanceolatus*. HFF315 (contig NODE_76626_length_699_cov_12.623794) was similar to reverse transcriptases from a bacterium in the family Sphingobacteriaceae and a jockey-like mobile element in a spider, *Araneus ventricosus*.

## Discussion

### FISH

FISH provided the most positive evidence of similarity between the S and E chromosomes. The count and arrangement of colored hybridization sites strongly indicated syntenic relationships between each of the E chromosomes and an S chromosome, and all four S chromosomes could be matched to one or another E chromosome. This result concurs with the findings of Staiber (2004) that the K chromosomes of *Acricotopus lucidus* share sequences with the S chromosomes of that species.

### AFLP

The complete identity of AFLP bands for all but one combination of enzymes and selective nucleotides is a second line of evidence in favor of evolution of E chromosomes from S chromosomes. The lone deviant band, which gave rise to sequences E1-1 and E1-2, appeared to represent non-overlapping parts of a reverse transcriptase (RNA-dependent DNA polymerase) from a retrotransposon. The reverse transcriptase is not necessarily functional and might have accumulated mutations subsequent to the last insertion event. The E-chromosomal variant of each, if there is one, appears to be very similar to one to three S-chromosomal variants, but it either contains an insertion or has moved a restriction site. The more distant hit to *Enterobacter cloacae* is possibly coincidental or indicative of a horizontal gene transfer between host and the bacterium. Long-read sequencing has found evidence of horizontal transfer of endosymbiotic bacterial sequence to insect genomes, up to a linearized complete genome of *Wolbachia* to *Drosophila ananassae* (Tvedte et al. 2022).

### SSH

Any sequences limited to E chromosomes would have appeared as extra spots with the ovarian probe. The complete identity of the spotting pattern with either probe, except for two extra spots with the non-ovarian probe, indicates that E chromosomes lack diagnostic sequences. However, the sensitivity of filter hybridization to differences in target content is surprisingly low for two reasons. First, isolated SNPs would not much affect the hybridization kinetics of long probes, so spots were not expected to respond to allelic differences between probe and target. Second, spot intensity depended on target copy number. To prevent oversaturation of the most repetitive spots, autoradiogram exposure was limited to too short a time to detect single-copy clones. There possibly was presence-absence variation of low-copy loci that went undetected. Furthermore, both the library clones and the probes were obtained from a metagenome that contained the Hessian fly and its resident microflora. Variation in microbial populations could affect visibility of clones derived from microbial sequences. The almost completely identical spot pattern with both probes indicates that the probes had equally sampled the microflora.

### Shotgun sequencing

Only two sequences, HFE174 and HFE315, could be amplified with the expected product size from ovaries only and not from heads. Neither HFE174 and HFE315 was amplified from all ovaries; both segregated somewhat or fully independently of each other, since both were amplified together from only two families. The presence in ovarian samples suggested that both reside in E chromosomes, but because of lineage sorting the observed segregation is not consistent with permanent presence of diverse E chromosomes in the population. It is possible that one or both are in fact bacterial sequences and that some Hessian flies fail to acquire the right bacteria, but this would require their annotated presence in spider and fish genomes to result from misassembly that incorporated bacterial sequences. It is also possible that some individual Hessian flies lose their germ line E chromosomes upon aging, even if this eliminates their reproduction, and that such individuals were sampled in some families.

The sequencing results support the preliminary conclusion of Zhao et al. (2015) that Hessian fly E chromosomes are very similar to S chromosomes.

Our shotgun strategy depended on the completeness of both Hessian fly assemblies, the similarity of both assemblies to the genome of biotype L investigated here, and the comprehensiveness of the microbial databases. In reality, each Hessian fly assembly contained sequences that were absent in the other assembly. A blastn search of Kansas Great Plain against Berkeley revealed 4779 contigs that were present only in Kansas Great Plain. The reciprocal search found 388 contigs that existed only in Berkeley. The Kansas Great Plain assembly was 26.5% larger than the Berkeley assembly. While it is likely that the two Hessian fly genomes are not exactly equal in size, it is also likely that neither assembly is complete, Kansas Great Plain is more complete, and that repetitive elements have been collapsed differently in each assembly. Similarly, many microbes remain uncultured and unrepresented in sequenced environmental samples, in addition to the potential for incompleteness even in small microbial genomes.

Even so, our set of almost 2.8 billion reads was deep enough to encounter all major families of repetitive elements in Hessian fly genomes. It is very unlikely that repetitive sequences specific to E chromosomes were missed entirely. Instead, it appears likely that E chromosomes differ allelically in critical places compared to their S homeologs. The initial filtering of sequences against the Kansas Great Plain genome could have eliminated such reads from further consideration. Similarly, the SSH protocol probably would have eliminated tester sequences that differed only by a SNP or two from driver sequences. Yet the proteins that regulate chromatin packing and centromeric binding to the mitotic spindle might be sensitive to single base variation in their DNA binding sites.

The similarity of S and E chromosomes suggests an alternative strategy to detect sequences that are enriched in E chromosomes, although possibly there are no such sequences. The strategy is to calculate depth of read coverage across the Kansas Great Plain genome separately for heads and ovaries, looking for sequences with “significantly” increased read depth in the latter. Even though the head and ovary libraries differ in size and sequencing depth, peaks in the ovary:head ratio would indicate E-enriched sequences if there were enough E chromosomes in the ovarian sample. An absence of peaks could result from insufficient amount of E-chromatin in the ovaries or an identical balance of repetitive element families in the S and E chromosomes.

It is conceptually possible in future research to analyze reads that overlap low-copy regions in the genome for sequence variants (SNPs, small indels) that are specific to gonads, thus likely specific to E chromosomes. Paralogous variants among low-copy regions in S chromosomes would appear in both heads and ovaries. Any ovary-specific variants in repetitive regions likewise would likely be specific to E chromosomes or at least enriched in E chromosomes. While sets of multiple SNPs can be affected by the production of chimeric reads because of self-priming of incomplete amplicons on the “wrong” allele during PCR, tallies of individual SNPs would remain reliable so long as sequencing depth sufficiently exceeds the frequency of sequencing errors.

Remote hybridization in grasses inspires a possible hypothesis on the origin of E chromosomes. Pollination of wheat (*Triticum aestivum*) and oat (*Avena sativa*) with maize (*Zea mays*) pollen sometimes yields embryos that can be cultured and grown out to maturity. Because of chromosome elimination during early embryogenesis, many of the resulting plants are maternal haploids, but some carry one or more maize chromosomes, and stable addition lines have been obtained upon chromosome doubling (Kynast et al. 2004). The maize chromosomes in these plants are heterochromatic with limited gene expression, even though they were fully active in the maize parent one to a few generations earlier. It is possible that the E chromosomes came into an ancestor of Hessian fly as a result of an analogous interspecific hybridization, while their maintenance would depend upon allele-specific and tissue-specific interaction with genes that regulate cell cycle and chromatin packaging.

The apparent collinearity of each E chromosome with an S chromosome, as demonstrated by FISH, contrasts with the structure reported by Kinsella et al. (2019) of the single germline restricted chromosome of the zebra finch (*Taeniopygia guttata*). The latter chromosome has been sequenced and bears genes from all the other chromosomes in apparently random order.

Finally, germ line specification in Hessian fly depends on a cytoplasmic structure termed an “oosome” (Bantock 1970; Kloc and Zagrodzinska 2001; Kloc et al. 2022; Quan et al. 2019), which accumulates transcripts of particular genes that apparently define the germ line. In an undisturbed early embryo, which is coenocytic, the germ line nuclei are in physical contact with the oosome. Physically separating the germ line nuclei from the oosome by centrifugation results in loss of all E chromosomes from the germ line. Physically bringing somatic nuclei into contact with the oosome results in retention of the E chromosomes in somatic tissues, although this deserves a critical, modern re-evaluation. Possibly the oosome locally affects the cell cycle in a way that allows inefficiently or insufficiently spindle-attached centromeres of E chromosomes to pass to the poles at mitotic anaphase. Thus, centromeric repeats are a promising place to look for E-specific sequence variants.

## Supplementary Information

Supplemental Figure 1. Ovary dissected from a third-instar, female Hessian fly larva. (PNG 1363 kb)

High Resolution Image (TIF 2815 kb)

Supplemental Figure 2. Collected ovaries before DNA extraction. (PNG 1434 kb)

High Resolution Image (TIF 4314 kb)

Supplementary file 1 (DOCX 31.0 kb)

## Data Availability

Wheat lines and Hessian fly biotypes can be provided upon reasonable request. Sequences exist in NCBI GenBank as indicated in Table 1 and Supplemental Table 3.
