# Supplementary figures and images for: Molecular characterization of eliminated chromosomes in Hessian fly (Mayetiola destructor (Say))
Source: Chromosome Res. 2023 Jan 24;31(1):3. doi: 10.1007/s10577-023-09718-8 (PMC9873768; doi:10.1007/s10577-023-09718-8)

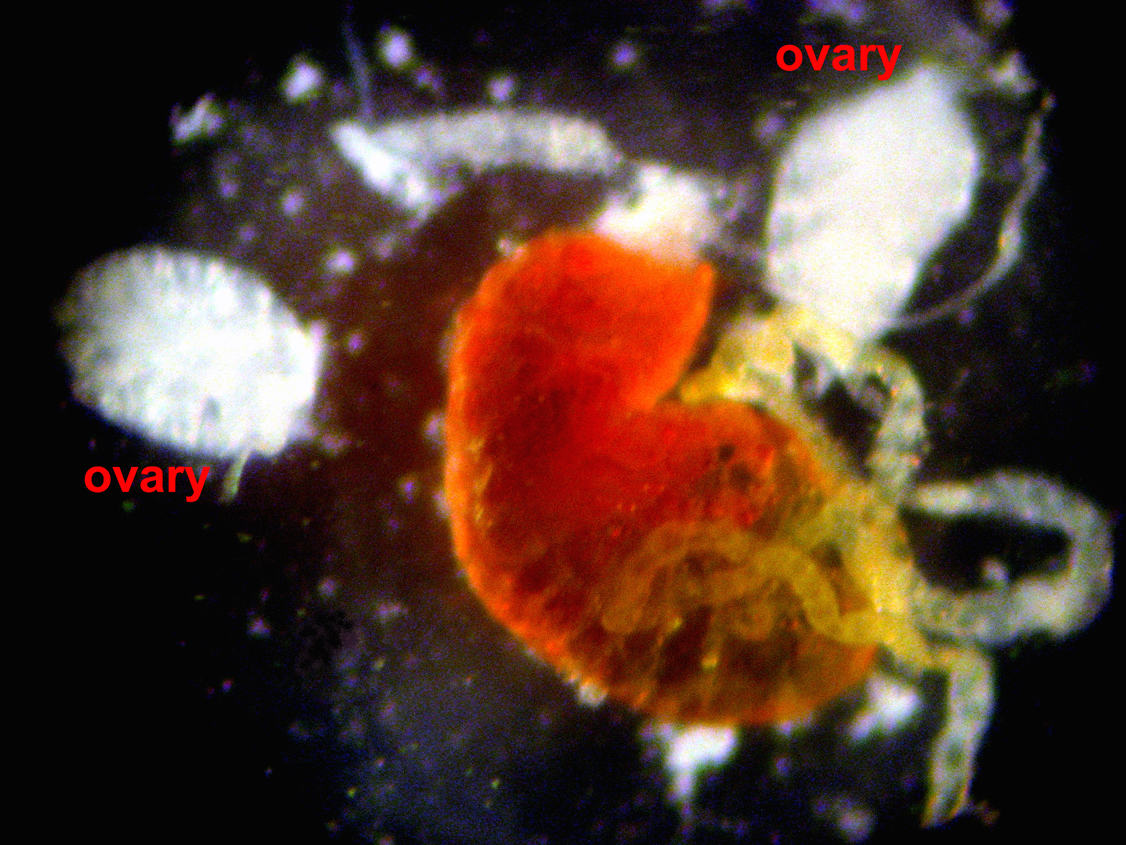

Supplement: Supplementary file 1 — Ovary dissected from a third-instar, female Hessian fly larva. (PNG 1363 kb) [file 10577_2023_9718_Fig8_ESM.png]

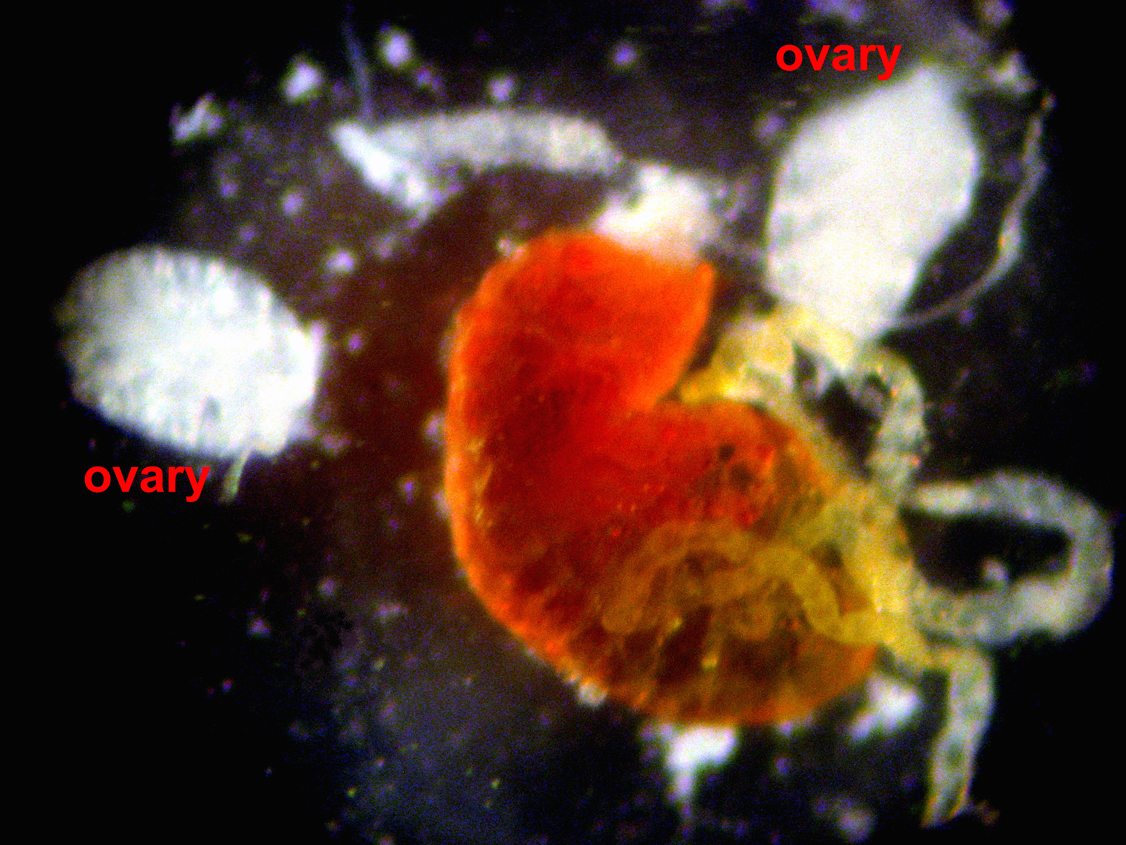

Supplement: Supplementary file 2 — High Resolution Image (TIF 2815 kb) [file 10577_2023_9718_MOESM1_ESM.tif]

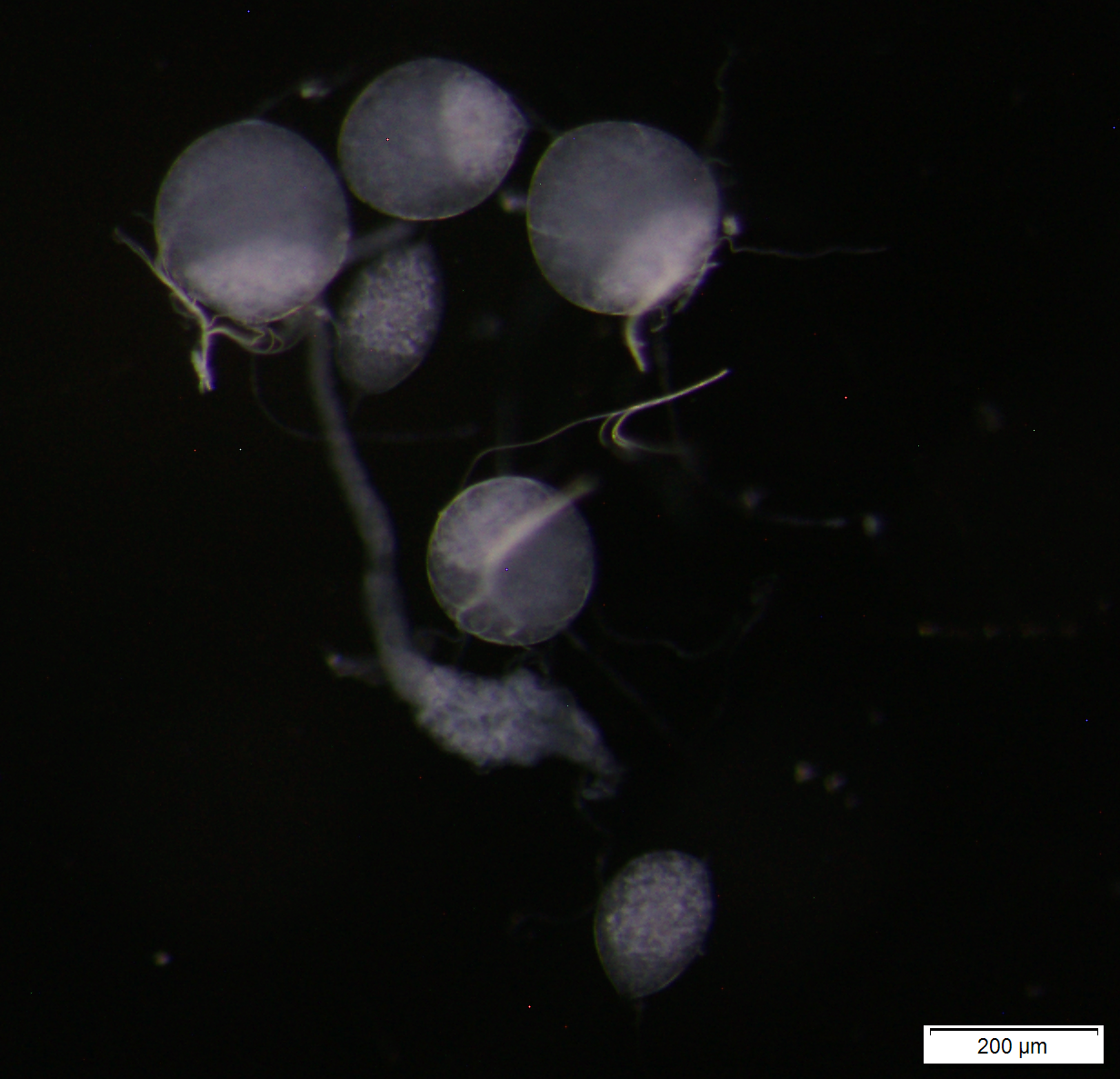

Supplement: Supplementary file 3 — Collected ovaries before DNA extraction. (PNG 1434 kb) [file 10577_2023_9718_Fig9_ESM.png]

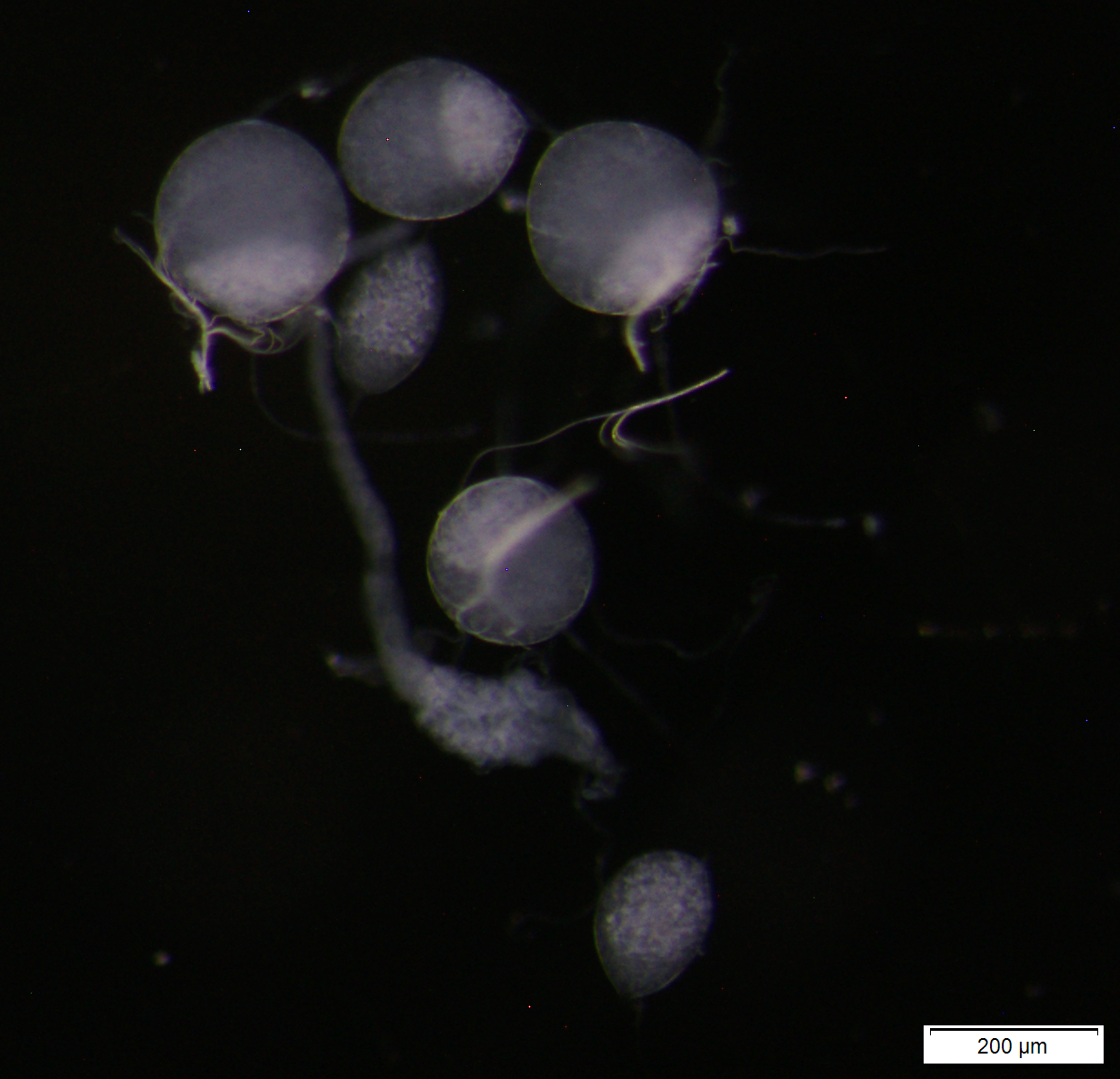

Supplement: Supplementary file 4 — High Resolution Image (TIF 4314 kb) [file 10577_2023_9718_MOESM2_ESM.tif]
