# Supplementary material for: Molecular characterization of eliminated chromosomes in Hessian fly (Mayetiola destructor (Say))
Source: Chromosome Res. 2023 Jan 24;31(1):3. doi: 10.1007/s10577-023-09718-8 (PMC9873768; doi:10.1007/s10577-023-09718-8)
Supplement: Supplementary file 5 — (DOCX 31.0 kb) [file 10577_2023_9718_MOESM3_ESM.docx]

Supplemental table 1. BAC DNA probes used for FISH

| Probe name | BAC location^a^ | Embedded SSR Primer (L) | Embedded SSR Primer (R) | Chromosome location | GenBank |
| --- | --- | --- | --- | --- | --- |
| Hf11g22^b^ | 11G22 | None | None | A1 NOR (yellow) |  |
| Hf32 | 1L8 | AAGAATGCAACGTGTGATGG | CAAATAGCCAAAGGCCACAT | A2 L (green) | EU340393 |
| Hf3 | 11L24 | ACTTTCGCCAAAGAAAACGA | CCAAACGAAACAAGCACAGA | X1S (red) | EU340371.1 |
| Hf11 | 6L2 | CAGGATGATGCGTGTCACAT | TCCGAAATATTTAGCTCAACCAA | X2L (blue) | EU340379.1 |

^a^Schemerhorn et al., 2009

^b^Hf11g22 provided by Dr. Jeff Stuart’s laboratory.

Supplemental table 2. Genome databases to which ovarian reads were aligned^a^.

| Source | Scaffold count | Taxon count | Description |
| --- | --- | --- | --- |
| GCA_001014435.1 | 28220 | 1 | Hessian fly, Berkeley assembly |
| GCA_000149185.1 | 24475 | 1 | Hessian fly, Kansas Great Plain assembly |
| GCA_000001405.28_GRCh38.p13 | 640 | 1 | Human (Homo sapiens) |
| iwgsc_refseqv2.1 | 22 | 1 | Wheat (Triticum aestivum cv. Chinese Spring) |
| Invertebrates | 3156641 | 267 | One genome per invertebrate taxon in ftp site |
| Protozoa | 216549 | 94 | One genome per protozoan taxon in ftp site |
| Fungi | 33923 | 421 | One genome per fungal taxon in ftp site |
| Bacteria | 2957055 | 39133 | One genome per bacterial taxon in ftp site |
| Archaea | 35943 | 930 | One genome per archaeal taxon in ftp site |
| Viruses | 14207 | 11229 | One genome per viral taxon in ftp site |

^a^One taxon was counted per “Directory” line in the index.html file resulting from wget –pr against the source at the NCBI ftp site, <ftp://130.14.250.11/genomes/refseq/> . The wheat genome came from <https://www.wheatgenome.org>.

Supplemental Table 3. Sequences of cloned AFLP bands possibly limited to E-chromosomes.

>E1-1

GACTGCGTACCAATTCAACACATTCGCTGATGAAACAAACAAATTGAAATCATTCATTAT

CCTTGGTATTTGCTGTGGCTCTGATGAGGAGAATATCACACATATTCGCAATACACTGGT

AAGCCACGGATGTCAGACGGGCTTCGATCTTGAACGTTTCACCACCGGATTCCAAAAACA

AAATCCTAAAAATATCAATTTACTGTATCGTATCACAGTCCCAAGCAATTTCGATGAGAA

ATTGTTTGCCAATGTACGAACAATTGGTGTCTTCGGCGTCCGCATCGAAAAAATGAACAA

TGGCAATATTGTGCAATGCAGAAACTGTCAACGTTTTTCCCACACGGCACGACAGTGCTA

CTTCGGATATCGTTGtGTTACTCAGGACTCATC

>E1-2

gactgcgtacCAATTCAATAAAATGAACGATAGACTTGAAAACGAACACATCAATCGTAT

TCAGAGTAGCATAAAGTCTGACCCGGCATCATTCTGGAAATTCGCAAAGGTTGATAGGTC

GTCAGAAACATATCCAACTGAAATGCACTACCTTGATAAAAATGGTGCAACACCACACGA

AATTGTTGATTTATTTGCAACATATTTTGAATCAACTTATGACACAGATGATTGGGACAG

CAATTTCAATGACACTTATCAATATGTTTCGAATTCGCATGAAGTCGATGTCACTCTGGG

TGATATTGAAATGGCAATAAATTCATTGAAAACGAAAGGTGGCATTGGACCTGATGAAGT

GTCTCCATATGTCATCAAAATGTGTGTTACTCAGGACTCATC
